# Effective *Striga* control and yield intensification on maize farms in western Kenya with N fertilizer and herbicide-resistant variety

**DOI:** 10.1016/j.fcr.2023.108924

**Published:** 2023-05-15

**Authors:** Dries Roobroeck, Geoffrey Kimutai, Fred Kanampiu, Wilson Ng’etich, Kristina Roing de Nowina, Bernard Vanlauwe

**Affiliations:** a International Institute of Tropical Agriculture (IITA), Nairobi, Kenya; b Department of Soil Science, University of Eldoret, Eldoret, Kenya; c GOPA Worldwide Consultants, Bad Homburg, Germany; d Department of Soil and Environment, Swedish University of Agricultural Sciences, Sweden; e System Management Office, Consultative Group of International Agricultural Research, Montpellier, France

**Keywords:** Weed control, Soil nutrient management, Imidazolinone resistant maize, Sustainable intensification

## Abstract

**Context:**

Maize production in western Kenya is limited by the spread of parasitic weed *Striga hermonthica* and depletion of soil nutrient stocks. Nitrogen (N) fertilizer and imidazolinone resistant (IR) maize are key elements in the agronomic toolbox to control infestations and enhance yields

**Research question:**

The circumstances under which their use, individually or combined, is most effective on farmer fields have not been well documented. Inappropriate management decisions and low returns on investments arise from this knowledge gap, causing hunger and poverty in smallholder communities to persist.

**Methods:**

Experiments were carried out on 60 fields in three different agroecosystems of western Kenya using full-factorial treatments with non-herbicide treated maize (DH) and herbicide treated maize (IR), and N fertilizer omission and application. Trials were stratified on a field with low and high soil fertility within individual farms and repeated over two seasons.

**Results:**

Cultivating IR maize instead of DH maize decreased the emergence of *Striga* with 13 shoots m^−2^ on average while applying N fertilizer on DH maize led to a reduction of 5 shoots m^−2^ on average. Decreases of *Striga* by use of IR maize and N fertilizer were between 6 and 23 shoots m^−2^ larger at the site with high levels of infestation than at the sites with medium or low emergence. Input of N fertilizer increased grain harvests by 0.59 ton ha^−1^ on average while use of IR maize enhanced the productivity with 0.33 ton ha^−1^ on average. Use of N fertilizer had similar yield effects in all three sites, whereas use of IR maize at the site with high *Striga* emergence increased maize production by 0.26–0.39 ton ha^−1^ more than at the sites with medium or low emergence.

**Conclusions:**

The greater *Striga* responses to IR maize and the greater yield responses to N fertilizer demonstrate their use could be optimized according to field conditions and management goals. Combining IR maize and N fertilizer has larger added yield benefits where their individual effects on grain productivity are smaller.

**Significance:**

Findings from this study indicate that farmers in western Kenya require guidance on how to align the use of herbicide resistant maize and inorganic N inputs with the level of *Striga* infestation and maize yield on their fields for effectively controlling the pernicious weed and enhancing food production.

## Highlights

• IR maize is more effective than N fertilizer in reducing *Striga* emergence.
• N fertilizer is more effective than IR maize toward increasing maize grain production.
• *Striga* emergence alone does not predict the yield gain by use of IR maize and/or N fertilizer.
• Yield benefits of combining N fertilizer and IR maize is linked to yield effects of individual practices under some conditions.

## 1 Introduction

The nutritional and financial security of several million people in small-scale farming communities from the mid-altitude highlands of western Kenya depends for a very large part on maize production. Multiple adverse factors however cause large deficits in actual grain yields of 2.2 ton ha^−1^ compared to attainable levels across most of the region (Muniola et al., 2019). Inappropriate use of improved varieties, mineral fertilizers, organic amendments, and best agronomic practices, coupled with poor and declining soil fertility are some of the main culprits (Okalebo et al., 2006, Tittonell et al., 2008b). The parasitic witchweed species *Striga hermonthica* is an important menace for maize crops in western Kenya (Atera et al., 2013) since it has spread to all growing areas helped by inadequate control measures (De Groote et al., 2010a). *Striga* spp. weeds siphon off valuable photosynthates from cereals by latching onto the roots (Okonkwo, 1966) and cause the crop to decrease stomatal conductance which leads to lower primary productivity (Frost et al., 1997). Grain yield losses up to 50% are common in western Kenya because of *Striga* spp. parasitism (Atera et al., 2013), putting households that gain food and income from maize farming in serious jeopardy.

Nitrogen (N) fertilizer is widely used to improve maize production and soil fertility on small-scale farms across the region and forms one of the biggest costs for producers. Some field studies in western Kenya found lower rates of *Striga* emergence when mineral N was applied (Odhiambo and Ransom, 1996, Jamil et al., 2012) but this has not been consistently confirmed. Imidazolinone resistant (IR) maize varieties with an herbicide seed coat that provides season-long control of *Striga* are marketed by many local dealerships. Grain yields of IR maize in western Kenya showed to be 0.4 ton ha^−1^ higher than for improved varieties without herbicide coating (Kanampiu et al., 2003). Farmer evaluations across the region found a strong preference for growing IR maize with fertilizer instead of common non-herbicide treated varieties without fertilizer (De Groote et al., 2010b). Applying inorganic N and herbicide coated seed must generate sufficiently large gains in productivity for farmers to recover their investments and be left with more money, but this is often not the case. Uncertainty about the yield benefits of management practices on *Striga* spp. infested croplands keeps small-scale producers from adopting them (Hearne, 2009). Therefore, practical advisories are needed that help decide how urea fertilizer and IR maize are best applied under heterogeneous conditions.

Large differences in the response of *Striga* emergence and maize productivity to improved agronomic practices are generally found between farming systems, growing seasons and individual fields. Soil properties like texture, nutrient availability, organic matter content and *Striga* seed bank have been shown to influence the *Striga* reduction and yield gain by use of N fertilizer and IR maize (Okalebo et al., 2006, Tittonell et al., 2012). Considerable seasonal variation may occur in the benefits from IR maize (Ransom et al., 2012) and N fertilizer due to their strong dependence on rainfall conditions. The interaction of the two practices can be influenced by soil and weather properties, determining if combined N fertilizer and IR maize gives rise to additional yield benefits or not. Knowing how *Striga* spp. and maize respond to use of N fertilizer and IR maize, separately or combined, at locations and times with different characteristics will help farmers make better decisions to combat the parasitic weed and increase crop productivity.

For this study, trials were set up on 60 smallholder fields in western Kenya over two consecutive growing seasons covering soil fertility gradients perceived by landowners. The experiment involves treatments without and with N fertilizer and/or IR maize that were randomly located on fields. Specific objectives were to assess patterns of *Striga* emergence and maize yield under the tested practices: 1) between three distinct growing areas, 2) among fields with low or high soil fertility, and 3) when separately or jointly utilized. Findings from our study provide insights on how to best use N fertilizer and IR maize for controlling the spread of *Striga* spp. and intensifying maize production on farms in western Kenya.

## 2 Materials and methods

### 2.1 Study design

The experiment was performed at three study areas of 100 km² located in Bondo, Siaya and Vihiga County characterized by differences in altitude and soil mineralogy (Table 1). A random selection of 10 *Striga* infested small-scale farms from each study area was made based on an earlier surveillance (Larsson, 2012). Within each farm the experiment was implemented on two separate fields, with high and low soil fertility as defined by farmer perceptions, giving a total of 60 for the study. Experimental trials were repeated during two consecutive growing seasons, short rains of 2011 (SR2011) and long rains of 2012 (LR2012), whilst keeping them on the same place in farmer fields. The study looked at four treatments that consist of two non-isogenic maize varieties, i.e., non-IR (DH04, Kenya Seed Company Ltd.) and IR (WS303, Western Seed Company Ltd.), and two fertilizer applications, i.e., 0 or 60 kg N ha^−1^. At the onset of every season the experimental fields were manually tilled up to 20 cm depth following farmer practice. Plots for individual treatments measured six by six meter, accommodating eight rows at a spacing of 75 cm and 24 stands of maize per row at 25 cm spacing. All plots had a one-meter buffer strip around them. Treatments with N fertilizer received Urea in two splits through a basal application of 25 kg N ha^−1^ at planting and a top-dressing of 35 kg N ha^−1^ at six weeks after planting (WAP). All treatments received a basal fertilizer application of 60 kg P ha^−1^ (triple superphosphate, TSP) and 60 kg K ha^−1^ (potassium chloride, KCl) at planting to avoid that their deficiencies would confound effects of N fertilization. Fertilizers were evenly distributed over all planting holes or stands with a calibrated dollop cup. Basal inputs of N, P and K were placed at about 10 cm depth and then covered with 5 cm of soil. Every planting hole was sown with two seeds before it was sealed with 5 cm of soil. Thinning and gap filling of stands was performed until 4 weeks after planting (WAP) to achieve the desired plant density. Weeds other than *Striga* were removed two times up to four WAP by cutting them at level surface.

**Table 1:** Geographic position and mineralogical classification of study sites, and total precipitation during growing seasons.

Study site		Bondo	Siaya	Vihiga
Center point (lat long)		0.24, 34.27	0.06, 34.29	0.05, 34.72
Elevation (masl) *		1331	1330	1463
Soil mineralogy^†^		ferralic Cambisol	haplic Acrisol	humic Acrisol
	**Season**			
Cumulative rainfall (mm)	SR2011	770	674	811
	LR2012	1158	994	1213

lat = latitude, long = longitude (decimal degrees); masl = meters above sea level, *Digital Elevation Model (Jarvis et al., 2008); † Soil Atlas of Africa (Jones et al., 2013).

### 2.2 Rainfall data

Information on the monthly amounts of precipitation received in each study area and growing season were retrieved from the Climate Hazards Group Infrared Precipitation database (Funk et al., 2014). Cumulative amounts of rainfall were calculated from 1 September 2011–29 February 2012 (SR2011), and 1 March to 31 August 2012 (LR2012).

### 2.3 Soil properties

Composite samples were taken of the top 20 cm soil in each treatment plot before the start of SR2011 by collecting material with an auger at the three random points. The *Striga* seedbank in a study area was determined for five experimental fields from each soil fertility class, making a total of 30 samples. Chemical soil properties in a study area were characterized for two fields from each soil fertility class, giving a total of 12 samples. Numbers of *Striga* seeds were quantified for 250 g of soil using elutriation and microscopic counting techniques (Eplee and Norris, 1987). The acidity (pH) of soils was determined with an electrode in the supernatant of 10 g soil that was mixed with distilled water (Mettler Toledo, USA). Soil organic carbon (SOC) was determined through sulphuric acid digestion of 20 g soil, potassium dichromate reaction and colorimetric reading (Anderson and Ingram, 1993). Total nitrogen (Total N) of soils was quantified by Kjeldahl digestion of 20 g soil and colorimetric reading (Anderson and Ingram, 1993). Soil extractable P was determined by sodium bicarbonate extraction of 10 g soil and colometric reading (Olsen et al., 1954). Exchangeable K, Ca and Mg were measured on ammonium acetate extracts (1 molar, pH 7) of 10 g soil using atomic absorption spectrometry (Anderson and Ingram, 1993). The mean value of soil properties across individual treatment plots in a farmer field was used for statistical analysis.

### 2.4 *Striga* emergence and maize yield

For each treatment the number of *Striga* shoots and productivity of maize grains were assessed within a net plot of 12 m² covering the four inner rows, leaving one meter from all sides. The cumulative *Striga* emergence was determined by enumerating shoots at 6, 8 and 10 WAP, uprooting the weed after each tally, and summing up the three counts. Maize crops were harvested when 75% of plants had dried and while in the field the total fresh weight of maize ears, without husks, was measured. Five maize ears that represent the range of sizes in each treatment plot were subsampled. The fresh weight of maize grains and cores was determined in the field after manually separating the two fractions. Grains and cores were placed in an oven at 65 °C for 48 h before taking the dry weight. Yields of maize grain were calculated using the fresh weight of all maize ears and the ratio of dry to fresh weight for grains and cores in the subsampled ears.

### 2.5 Statistical analysis

All statistical computing and graphic design for this paper was carried out in R software, version 3.3.2 (R Development Core Team, 2016). Linear mixed modeling was performed in the ‘lme4′ package, while ordinary linear modeling in the ‘stats’ package. Residual normal distribution and homoscedasticity of each model were ascertained visually based on plotting residuals against theoretical quantiles and fitted values. Data on *Striga* seedbank and emergence had to be transformed by its natural logarithm (Y = LogX) for achieving equal residual variance of fitted values. The variation in *Striga* seedbank and chemical properties of soils between the study areas and soil fertility classes was assessed using mixed models with their main and interactive effects, and individual fields as random intercepts. Comparisons of *Striga* emergence and maize grain yields were made in a mixed model with main and interactive effects for N fertilizer, maize variety and study area without and with nesting in fertility class, random intercepts for individual field trials, and random slopes for growing seasons. Responses of *Striga* emergence and grain yield were computed for the 60 N-DH, 0 N-IR and 60 N-IR treatments as compared to the 0 N-DH treatment (control). Differences of weed and crop responses were analyzed in mixed models with the main and interactive effects for treatment effects and study area without and with nesting in fertility class, random intercepts for individual field trials, and random slopes for growing seasons. The decision to exclude a main effect for the long and short rainy seasons was informed by a lack of significant differences in *Striga* emergence, maize yield and their responses between treatments, rather they are represented in the model as an analog for repeated measures. Significance testing of main effects and their interactions was performed through Type III analysis of variance with Satterthwaite approximation for degrees of freedom. Pairwise comparisons of means were made by least-squares with confidence intervals and standard errors of difference using the ‘lsmeans’ package. Least significant differences for each model were determined using the residual error of the model and degrees of freedom within groups. The yield gain ascribed to the interaction of N fertilizer and IR maize was calculated by subtracting the sum of the responses to the separate treatments over the control (0 N-DH) from the response to the combined treatments. Ordinary linear regressions between the individual effects of N fertilizer or IR maize on grain yield and the interaction of the two treatments were tested for each study area using data from the two growing seasons.

## 3 Results

### 3.1 Soil properties

The *Striga* seedbank in Bondo and Siaya was significantly higher than in Vihiga (Table 2). Fields from the low fertility class had a significantly higher *Striga* seedbank than those from the high fertility class in Siaya, but not in the two other study areas. The pH of soils in Bondo was significantly higher than in Siaya and Vihiga. SOC levels varied significantly between all study sites, being highest in Bondo and lowest in Vihiga. The pH and SOC did not show significant differences between the two fertility classes at any of the study sites. Total N levels of soils in Bondo were significantly lower than in Siaya and Vihiga. Fields from the low fertility class had significantly lower Total N than those of the high fertility class in Siaya, but not in the two other study areas. Extractable P and K contents of soils did not show any significant differences between the study areas and fertility classes. Exchangeable levels of Ca and Mg in soils varied significantly for each of the study areas. There were no significant differences in exchangeable Ca and Mg between the fertility classes at any of the study areas.

**Table 2:** Soil properties of subsampled farmer fields in each study site and fertility class at the start of the experiment (SR2011). Values are means and deviations of fields.

Study site	Bondo		Siaya		Vihiga	
Fertility class	Low	High	Low	High	Low	High
*Striga* seeds (kg^−1^)	33.4 ± 20.5	27.4 ± 23.1	58.2 ± 45.8	28.2 ± 21.4	5.80 ± 9.43	4.26 ± 5.87
pH_water_	6.0 ± 0.3	6.7 ± 0.5	5.2 ± 0.4	5.7 ± 0.8	5.3 ± 0.1	5.4 ± 0.1
SOC (%)	1.96 ± 0.35	2.21 ± 0.29	1.75 ± 0.16	1.84 ± 0.16	1.50 ± 0.03	1.59 ± 0.05
Total N (%)	0.15 ± 0.02	0.15 ± 0.04	0.13 ± 0.02	0.16 ± 0.01	0.13 ± 0.01	0.12 ± 0.01
P (mg kg^−1^)	2.52 ± 1.82	3.85 ± 1.03	5.29 ± 3.29	7.25 ± 3.20	3.24 ± 3.54	4.62 ± 3.98
K (cmolc kg^−1^)	0.37 ± 0.12	0.41 ± 0.11	0.27 ± 0.11	0.44 ± 0.23	0.13 ± 0.05	0.21 ± 0.11
Ca (cmolc kg^−1^)	6.41 ± 0.54	7.58 ± 0.35	1.20 ± 0.83	1.58 ± 1.15	4.62 ± 0.68	5.42 ± 1.34
Mg (cmolc kg^−1^)	0.28 ± 0.02	0.39 ± 0.10	0.56 ± 0.34	0.91 ± 0.33	1.16 ± 0.09	1.42 ± 0.17

### 3.2 *Striga* emergence

**Fig. 1:**
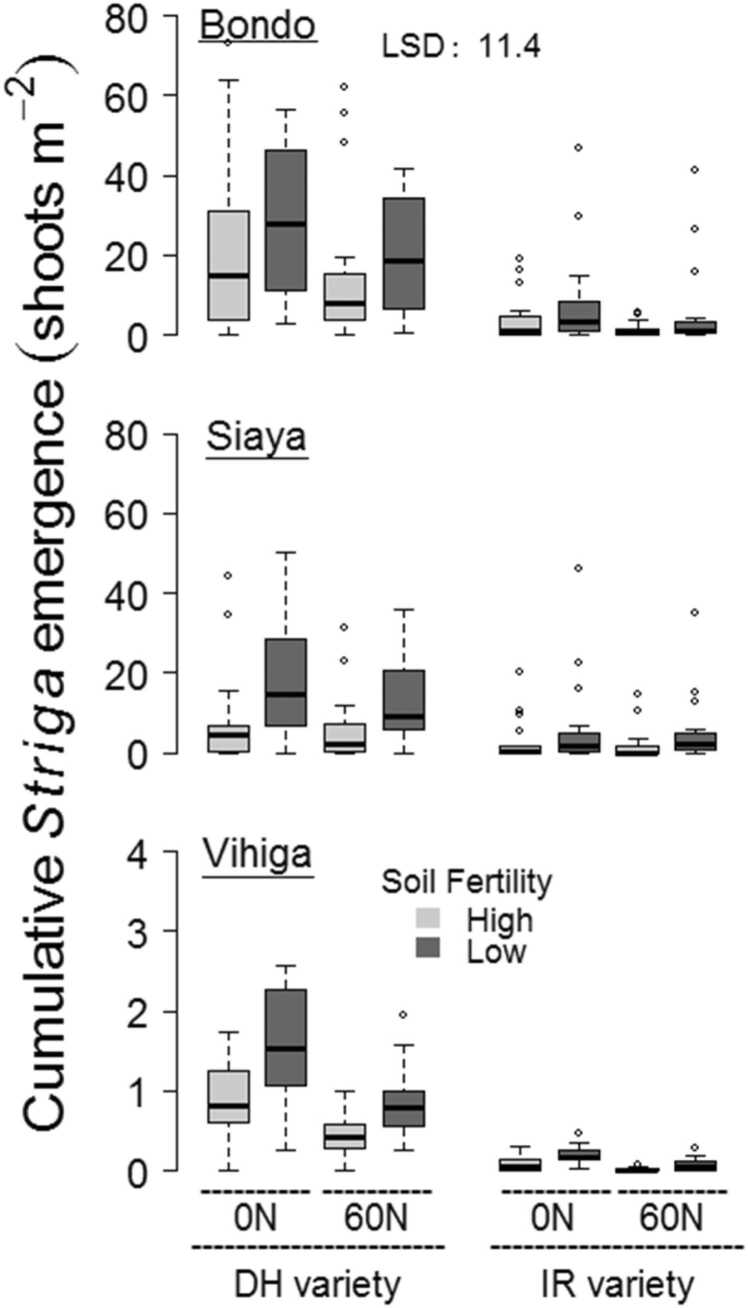
Boxplots with cumulative emergence of *Striga* in each study site and soil fertility class for different treatments with N fertilizer (0 N, 60 N) and maize varieties (DH, IR). Results from both growing seasons are included. The least significant difference (LSD) for predicted means from the mixed model is given.

Under the 0 N-DH and 60 N-DH treatment *Striga* counts varied significantly between all the study areas, measuring highest in Bondo and lowest in Vihiga (Fig. 1). Fields from the low fertility class had significantly greater *Striga* emergence under the 0 N-DH and 60 N-DH treatment than those from the high fertility class in Bondo and Siaya, but not in Vihiga. *Striga* counts for the 60 N-DH treatment were on average 0.44–13 shoots m^−2^ lower than for the 0 N-DH treatment across the study areas and soil fertility classes, but only in the low fertility class from Bondo was the difference significant. Under the 0 N-IR and 60 N-IR treatment *Striga* counts were significantly greater in Bondo and Siaya than in Vihiga. Under these two treatments, no significant differences in *Striga* emergence occurred between the two fertility classes at any of the study areas. *Striga* counts for 0 N-IR and 60 N-IR treatment were significantly lower than for the 0 N-DH treatment in each study area and soil fertility class, with decreases measuring 0.79–36 shoots m^−2^ and 0.86–39 shoots m^−2^ respectively. The effects of using N fertilizer or IR maize on *Striga* emergence did not significantly interact.

### 3.3 *Striga* responses

Decreases of *Striga* counts by N application for the non-herbicide coated DH maize varied significantly between each study area, measuring on average 5.9 and 9.1 shoots m^−2^ higher in Bondo than Siaya and Vihiga. No significant difference in reductions of *Striga* emergence by N addition to DH maize were found among the two fertility classes in any of the study areas. Decreases of *Striga* counts from using herbicide coated IR maize without N fertilization varied significantly between all study areas, measuring on average 14 and 23 shoots m^−2^ higher in Bondo than in Siaya and Vihiga. Switching to IR maize without N addition lead to significantly larger reductions in *Striga* emergence for the low fertility class than for the high fertility class in Bondo and Siaya, but not in Vihiga. The responses of Striga counts to N fertilizer input on the IR variety were not significantly different between any of the study areas and fertility classes. Decreases of *Striga* counts between the 0 N-DH and 60 N-IR treatment varied significantly between all study areas, measuring on average 16 and 25 shoots m^−2^ higher in Bondo than in Siaya and Vihiga. Use of N fertilizer and IR maize resulted in significantly larger decreases of *Striga* emergence on fields from the low fertility class than the high fertility class in Bondo and Siaya, but not in Vihiga.

Reductions of *Striga* counts by using the IR variety without N fertilizer were significantly higher than by using N fertilizer on the DH variety for the low fertility class in Bondo and Siaya. Decreases of *Striga* emergence between the 0 N-DH and 60 N-IR treatment were significantly larger than between the 0 N-DH and 60 N-DH treatment for both fertility classes in Bondo and the low fertility class in Siaya and Vihiga. Reductions of *Striga* counts between the 0 N-DH and 60 N-IR treatment were not significantly different from those between the 0 N-DH and 0 N-IR treatment for study areas and fertility classes.

### 3.4 Maize grain yield

**Fig. 2:**
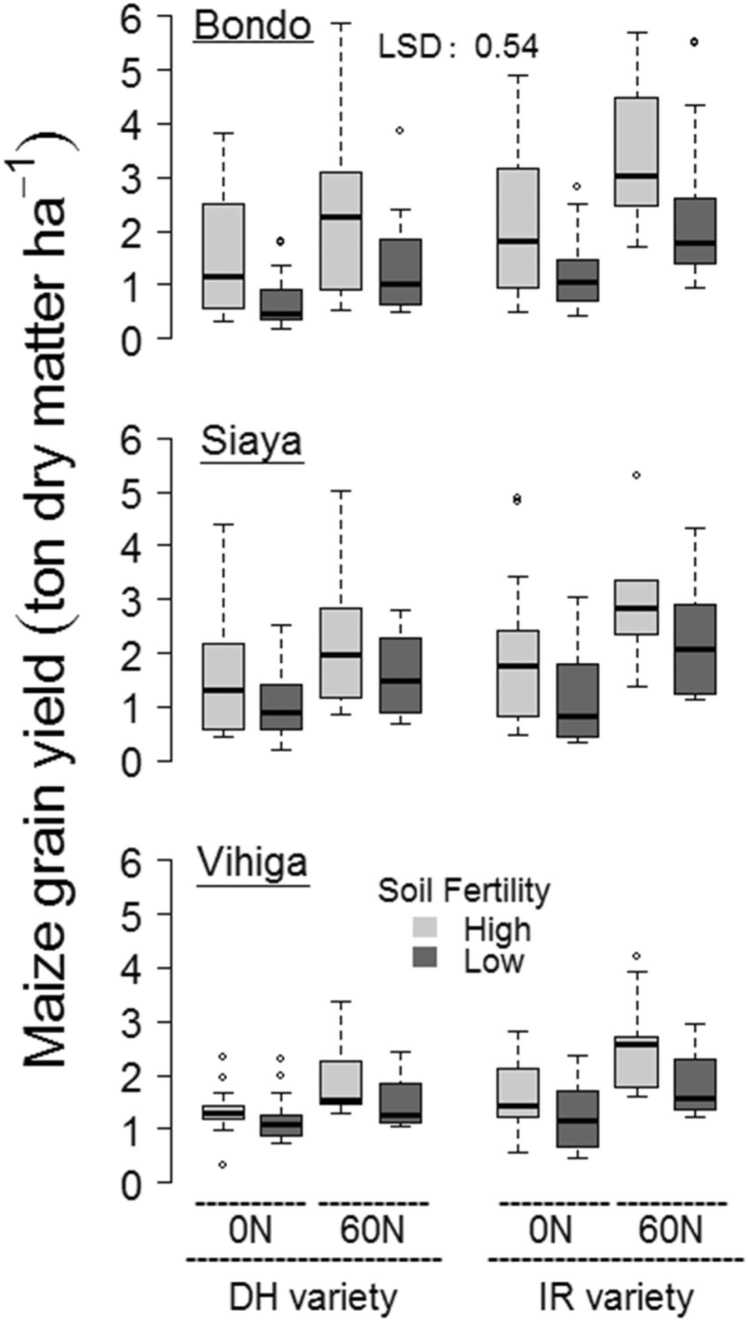
Boxplots with maize grain yields in each study site and fertility class for different treatments with N fertilizer (0 N, 60 N) and maize varieties (DH, IR). Results from both growing seasons are included. The least significant difference (LSD) for predicted means from the mixed model is given.

Under the 0 N-DH treatment, the productivity of maize grain was significantly larger in Siaya and Vihiga than in Bondo (Fig. 2). No significant differences in the maize yield occurred between the fertility classes under the 0 N-DH treatment at any of the study areas. Grain productivity under the 60 N-DH treatment was significantly higher in Siaya than Bondo. A significantly lower yield was recorded for the 60 N-DH treatment in the low fertility class than the high fertility class in Bondo, but not in the two other study areas. Maize productivity for the 60 N-DH treatment was on average 0.32–0.88 ton ha^−1^ higher than for the 0 N-DH treatment across the study areas and soil fertility classes, with significant differences for all except the low fertility class in Vihiga. Under the 0 N-IR treatment, there were no significant difference in maize yield between the study areas. The grain productivity of 0 N-IR treatment for the low fertility class measured significantly lower than for the high fertility class in Siaya, but not at the other study sites. Maize yields for the 0 N-IR treatment were on average 0.03–0.61 ton ha^−1^ higher than for the 0 N-DH treatment, with significant difference for both fertility classes in Bondo and only the high fertility class in Siaya.

Under the 60 N-IR treatment, the grain productivity was significantly larger in Vihiga than in Siaya. A significantly lower yield was recorded for the 60 N-IR treatment in the low fertility class than the high fertility class in Bondo and Siaya, but not in Vihiga. The increase of grain yields between the 0 N-DH and 60 N-IR treatment was significant in all study areas and soil fertility classes, ranging from 0.70 to 1.86 ton ha^−1^. Effects of using N fertilizer or IR maize on grain productivity had a significant positive interaction.

### 3.5 Maize yield responses

The increase of grain productivity between the 0 N-DH and 60 N-DH treatment did not significantly vary for the study areas, measuring on average 0.16 and 0.27 ton ha^−1^ larger in Bondo than in Siaya and Vihiga. No significant differences in yield gains by N addition to DH maize were found among the two fertility classes at any of the study areas. The increase of maize production between the 0 N-DH and 0 N-IR treatment was significantly larger than in Bonda than Vihiga by 0.39 ton ha^−1^ on average. No significant differences in yield gains by switching to IR maize without N addition were found among the two fertility classes in any of the study areas. Grain productivity increases between the 0 N-DH and 60 N-DH treatment were not significantly different from those between the 0 N-DH and 0 N-IR treatment for any of the fertility classes at the study areas. The average yield gain between the 0 N-DH and 60 N-IR treatment measured significantly higher in Vihiga than in Bondo and Siaya, by 0.75 and 0.46 ton ha^−1^ respectively. Increases of maize productivity by use of N fertilizer and IR maize were significantly lower for farmer fields from the low fertility class than the high fertility class in Siaya and Vihiga, but not in Bondo. Yield gains between the 0 N-DH and 60 N-IR treatment were 0.38–1.1 ton ha^−1^ higher than between the 0 N-DH and 60 N-DH treatment for individual fertility classes at the study areas, with significant differences expect for the low fertility class in Vihiga. Responses of maize productivity between the 0 N-DH and 60 N-IR treatment were significantly higher than between the 0 N-DH and 0 N-IR treatment in all fertility classes and study areas by 0.67–1.3 ton ha^−1^ on average.

### 3.6 Mutual benefits on maize grain yield

**Fig. 3:**
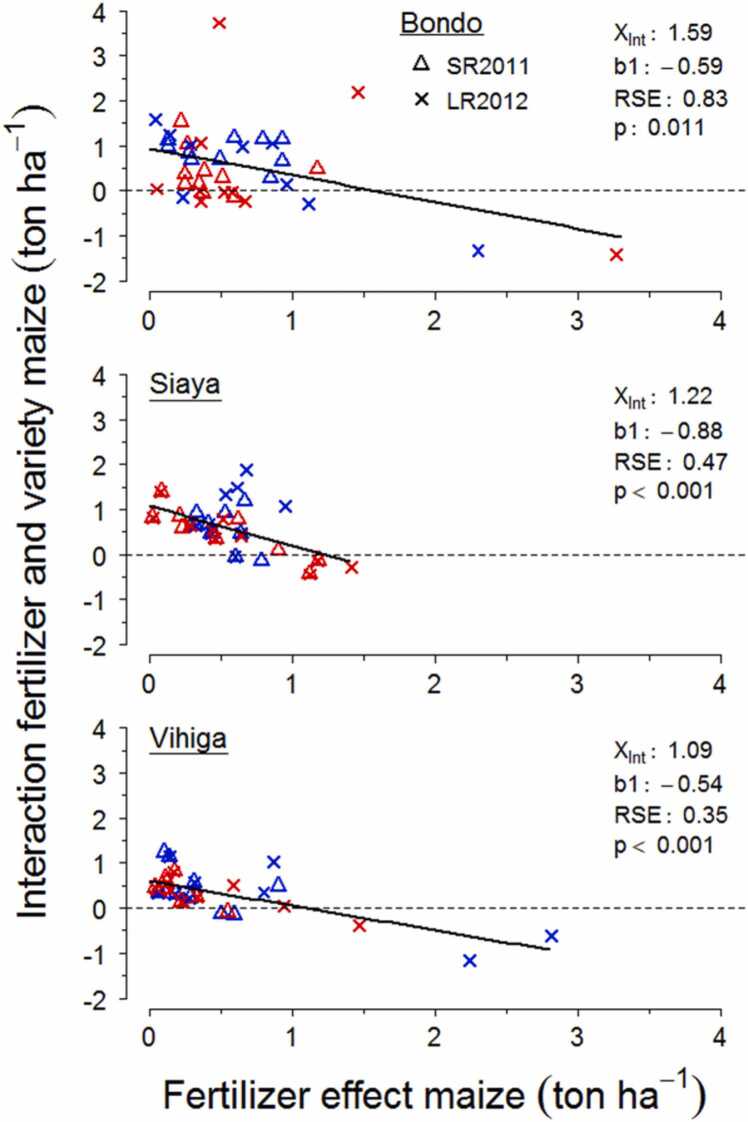
Relations of the yield benefit by N input to DH maize and the interaction between fertilizer and variety effects on productivity across individual farmer fields and growing seasons in each study site. Red and blue symbols represent the low and high fertility class. The intercept on the X axis (X_Int_), slope (b1), residual error (RSE) and significance (p) of regressions are shown.

Interactive effects of using N fertilizer and the IR variety on grain productivity were significantly decreasing with increasing response to N addition of DH maize increased on farmer fields for each study area (Fig. 3). In other words, the added benefit of using IR maize on top of N fertilizer was higher when the yield gain from adding N to DH maize was lower. The negative relations indicate that the yield effect of combining the two interventions does not proportionally increase with the yield effect of applying N. Interactions between N fertilizer and IR maize respectively shifted from positive to negative when the N fertilizer response of grain productivity from DH maize exceeded 1.59 ton ha^−1^ in Bondo, 1.22 ton ha^−1^ in Siaya and 1.09 ton ha^−1^ in Vihiga. There was large variability around the negative regression in Bondo since multiple farmer fields with low responses to N addition showed negative added benefits of IR maize, which was not the case for Siaya and Vihiga. No differences were found in the relationship between the interactive effect of the two treatments and N fertilizer responses for the two growing seasons at any of the study areas. Farmer fields from the low fertility class in Siaya exhibited a stronger negative relationship than their high soil fertility counterparts, while there were no such pronounced differences in Bondo and Vihiga.

**Fig. 4:**
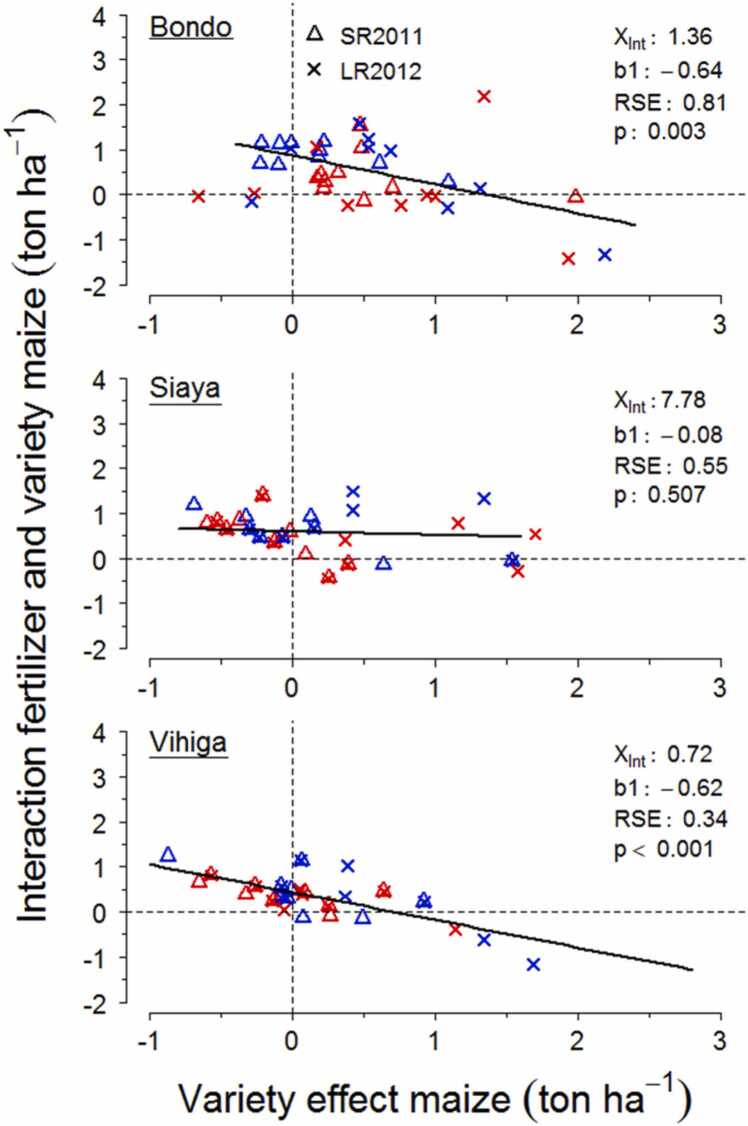
Relations of the yield benefit by switching from DH to IR maize without N input and the interaction between fertilizer and variety effects on productivity across individual farmer fields and growing seasons in each study site. Red and blue symbols represent the low and high fertility class. The intercept on the X axis (X_Int_), slope (b1), residual error (RSE) and significance (p) of regressions are shown.

The interaction between the effects of N fertilizer and IR maize on grain productivity was significantly decreasing when the response to use of IR maize without N addition increased on farmer fields in Bondo and Vihiga (Fig. 4). In other words, the added benefits of using N fertilizer on top of IR maize at these two study areas was higher when the yield gain from switching to IR maize in absence of N input was lower. The negative relations indicate that the yield effect of combining the two interventions does not proportionally increase with the yield effect of switching to IR maize. Change-overs from positive to negative interaction took place when yield gains from IR maize exceeded 1.36 ton ha^−1^ in Bondo, and 0.72 ton ha^−1^ in Vihiga. No significant slope in this relationship was shown for Siaya which means that the added benefit of using N fertilizer on top of IR maize remains unchanged with increasing yield gains from switching to IR maize in absence of N input. No marked differences were found in the relationship between the interactive effect of the two treatments and responses to IR maize for the two growing seasons and soil fertility classes at any of the study areas.

## 4 Discussion

In the first place, this study reiterates that use of N fertilizer and IR maize, separately or combined, has different contributions towards reducing *Striga* emergence and enhancing grain productivity on maize croplands of western Kenya. Addition of N fertilizer records consistently smaller reductions in *Striga* emergence than use of herbicide-coated maize, i.e., treatment effects differing by 0.56–20.7 shoots m^2^ within study sites. Input of N fertilizer always led to larger increases of grain yield than use of herbicide-coated maize, i.e., treatment effects differing by 0.19–0.29 ton ha^−1^ within study sites. Co-applying N fertilizer and IR variety did not give rise to significant reductions of *Striga* emergence compared to the use of herbicide-coated maize alone, but resulted in significantly higher yields than when the practices were use separately for all study sites. As for the representativity of our observations, any effect from the genetic differences between the maize varieties is arguable small as yields were similar in Vihiga where *Striga* pressure is very low and herbicide coating plays less of role. It must be emphasized that P and K fertilizer input rates are 2 and 1.5 times higher than what smallholder farmers generally use, so effects of N application reflect the attainable response and not the true-to-world situation.

Results of our study closely align with earlier findings in western Kenya and SSA that showed how N fertilization of maize without herbicide seed-coating is more effective towards increasing grain production than reducing *Striga* spp. emergence (Kanampiu et al., 2003). In the same paper these authors noted that switching to IR maize under low rates of N fertilizer was more effective towards decreasing *Striga* counts than enhancing maize yields. Obviously, the contrasting effects of N fertilizer and IR maize are linked to the different ways in which these combat *Striga* spp. and promote crop growth. Soil N input reduces *Striga* infestation by causing maize roots to exude less strigolactone compounds that stimulate germination of the parasite and guide it to the crop (Jamil et al., 2012). Likewise, intercropping maize with soybean can reduce *Striga* spp. emergence and enhance grain yields owing to increased levels of N in soils (Odhiambo et al., 2011). IR maize follows a different weed control mechanism, directly killing off germinated *Striga* spp. around the roots of the crop by targeted herbicide release through its seed coating (De Groote et al., 2007, Ransom et al., 2012). Cultivating IR maize is also known to significantly reduce the *Striga* seedbank of farms in western Kenya which aids in longer-term control (Vanlauwe et al., 2008). Grain yield increases by application of N fertilizer are ascribed to a greater nutrient availability in soils, and improved uptake through more vigorous root growth and branching of the crop (Pasley et al., 2019). Productivity gains from use of IR maize conversely result from avoiding that nutrients are siphoned off by *Striga* spp. (Aflakpui et al., 1998). The larger yield responses to N addition for IR maize than for non-herbicide coated maize we observed confirms that controlling *Striga* spp. enhances nutrient use efficiency.

In the second place, findings show that the use of N fertilizer and herbicide coated maize realize greater reductions of the parasitic weed on croplands where there is a higher level of infestation, such as in Bondo and Siaya or low fertility fields. Earlier research in western Kenya also found greater *Striga* spp. reductions by use of herbicide-coated IR maize for agroecosystems that suffer from a higher level of weed emergence (Kanampiu et al., 2018). Still, when there is low emergence without N input and IR maize, such as in Vihiga, the management practices can bring down *Striga* counts to less than 1 shoot m^−2^ or even zero, unlike is the case when infestations are high. Large similarities in proportional *Striga* reductions were moreover demonstrated among the study areas, i.e., 31–90% for Bondo, 32–79% for Siaya and 48–98% for Vihiga, which implies that actual differences in weed response are connected to the levels of emergence and size of seedbanks rather than the efficacy of herbicide coating.

Grain yield increases by use of N fertilizer and/or IR maize also showed strong dependence on the *Striga* infestation level and the soil fertility conditions of farms. These influences are visible from the similar or higher productivity gains by N addition on non-herbicide coated maize in Bondo and Siaya than in Vihiga despite the lower *Striga* emergence at the latter study area. Results for non-herbicide coated maize point at possible limitations of yield responses to N fertilizer by lower pH, organic carbon content and availability of K in Vihiga. At their turn, the higher yield increases from use of IR maize without and with N fertilizer in Bondo and Siaya than in Vihiga were linked to greater *Striga* emergence for non-herbicide coated maize without N fertilizer application, larger decreases of weed counts by the practices and better soil properties. The higher productivity gains from joint use of N input and IR maize for the high fertility class as compared the low fertility class at Siaya and Vihiga were conversely tied to lower *Striga* emergence and better soil conditions. Results from Vihiga where N input led to higher yield increases for herbicide coated maize than for non-herbicide coated maize confirm that herbicide-coated seed can improve nutrient availability on croplands with low *Striga* infestation levels. These insights underline the importance of informing agro-input dealers, extension agents and farmers in western Kenya about the variable productivity gains from use of N fertilizer and/or IR maize between agricultural systems and individual croplands with different characteristics.

Finally, our study illustrates that the relationships between yield increases from the individual practices and their interaction can provide guidance as to when combining N fertilizer and IR maize offers the largest complementary benefits. Where negative relations were found it means the two practices reinforce one another if there are low responses to N fertilizer alone due to siphoning of assimilates by *Striga*, and/or if there are low responses to IR maize alone due to insufficient availability of N. The negative regressions show that the two practices are not reinforcing each other on farms where use of urea fertilizer or herbicide-coated seed alone leads to a high yield gain. In Siaya, it appears that widespread limitation of N availability in soils alongside high levels of *Striga* infestation cause the use of urea fertilizer to generate complementary benefits across the entire board of productivity gains from herbicide-coated seed alone. The lack of significant relationships between yield responses and emergence of *Striga* for non-herbicide coated maize or corresponding response (results not shown) furthermore indicates that the effectiveness of N fertilizer and/or IR maize is affected more by nutrient availability than the level of parasitic weed infestation. It should be noted that the derived change-over points may be inaccurate given the small sample population and low number of fields with high responses to individual treatments. Hence, the change-over values and differences between sites that are presented in this paper should not be used as absolute indicators. Generally, farmers in the studied maize growing areas from Bondo, Vihiga and Siaya are thus best of combining both practices on *Striga* infested croplands where the yield response to urea application or herbicide-coated seed alone is lowest. As larger profits are made from the joint use of the two practices on worse off fields this extra money can enable farmers to gradually purchase N fertilizer or IR maize for the whole farm. If farmers can afford both inputs for all their maize fields, they will enhance maize production and build off *Striga* infestation faster. Optimizing the choice of crop variety and nutrient management is a common notion among integrated approaches for preventing *Striga* parasitism and increasing yields (e.g., Midega et al., 2014; Tadesse et al., 2018; Abdullahi et al., 2022). Knowledge about the effectiveness of individual and combined practices under varying conditions, as drawn from this study, is crucial to guide such decision processes. While the significant relationships we identified are valuable in advancing this field, they must be studied more extensively and mechanistically, as well as validated in other agro-climatic regions.

## 5 Conclusion

The potential benefits of strategic use of N fertilizer and/or IR maize observed in this study emphasize the need for integrated approaches to halt the spread of *Striga* spp. and intensify maize production in western Kenya. Insights from our study can help farmers, extensionist and agro-dealers in distinguishing where use of N fertilizer and herbicide-coated maize, separately or combined, is most effective towards controlling *Striga* spp. and enhancing maize yields. The differences in responses of the parasitic weed and cereal crop between agro-ecosystems and individual croplands provide a basis to formulate advisories for use of N fertilizer and IR maize. In specific, our study indicates that *Striga* spp. emergence for common non-herbicide coated maize, soil fertility perception of farmers and yield benefits from the individual practices can be used to guide improved management practices.

## Declaration of Competing Interest

The authors declare that they have no known competing financial interests or personal relationships that could have appeared to influence the work reported in this paper.

## Data Availability

Data will be made available on request.
